# Supplementary material for: Putting cumulative (dis)advantages in context: Comparing the role of educational inequality in later-life functional health trajectories in England and Germany
Source: PLoS One. 2020 Dec 30;15(12):e0244371. doi: 10.1371/journal.pone.0244371 (PMC7773250; doi:10.1371/journal.pone.0244371)
Supplement: S1 File — (DOCX) [file pone.0244371.s001.docx]

Online supplement to: Putting cumulative (dis)advantages in context: Comparing the role of educational inequality in later-life functional health trajectories in England and Germany.

*S1 Table: Representation of interview year and age for different cohorts*

| **Age in 2004** | **Birth year** | **Cohort** |  | **Observations at different ages over life course** | | | | | | |  |  |  |  |  |  |  |  |  |  |  |  |  |  |
| --- | --- | --- | --- | --- | --- | --- | --- | --- | --- | --- | --- | --- | --- | --- | --- | --- | --- | --- | --- | --- | --- | --- | --- | --- |
|  |  |  |  | 50 | 52 | 54 | 56 | 58 | 60 | 62 | 64 | 66 | 68 | 70 | 72 | 74 | 76 | 78 | 80 | 82 | 84 | 86 | 88 | 90 |
| 40 | 1964 | 5 |  | 2014 |  |  |  |  |  |  |  |  |  |  |  |  |  |  |  |  |  |  |  |  |
| 42 | 1962 | 5 |  | 2012 | 2014 |  |  |  |  |  |  |  |  |  |  |  |  |  |  |  |  |  |  |  |
| 44 | 1960 | 5 |  | 2010 | 2012 | 2014 |  |  |  |  |  |  |  |  |  |  |  |  |  |  |  |  |  |  |
| 46 | 1958 | 5 |  |  | 2010 | 2012 | 2014 |  |  |  |  |  |  |  |  |  |  |  |  |  |  |  |  |  |
| 48 | 1956 | 5 |  | 2006 |  | 2010 | 2012 | 2014 |  |  |  |  |  |  |  |  |  |  |  |  |  |  |  |  |
| 50 | 1954 | 4 |  | 2004 | 2006 |  | 2010 | 2012 | 2014 |  |  |  |  |  |  |  |  |  |  |  |  |  |  |  |
| 52 | 1952 | 4 |  |  | 2004 | 2006 |  | 2010 | 2012 | 2014 |  |  |  |  |  |  |  |  |  |  |  |  |  |  |
| 54 | 1950 | 4 |  |  |  | 2004 | 2006 |  | 2010 | 2012 | 2014 |  |  |  |  |  |  |  |  |  |  |  |  |  |
| 56 | 1948 | 4 |  |  |  |  | 2004 | 2006 |  | 2010 | 2012 | 2014 |  |  |  |  |  |  |  |  |  |  |  |  |
| 58 | 1946 | 3 |  |  |  |  |  | 2004 | 2006 |  | 2010 | 2012 | 2014 |  |  |  |  |  |  |  |  |  |  |  |
| 60 | 1944 | 3 |  |  |  |  |  |  | 2004 | 2006 |  | 2010 | 2012 | 2014 |  |  |  |  |  |  |  |  |  |  |
| 62 | 1942 | 3 |  |  |  |  |  |  |  | 2004 | 2006 |  | 2010 | 2012 | 2014 |  |  |  |  |  |  |  |  |  |
| 64 | 1940 | 3 |  |  |  |  |  |  |  |  | 2004 | 2006 |  | 2010 | 2012 | 2014 |  |  |  |  |  |  |  |  |
| 66 | 1938 | 2 |  |  |  |  |  |  |  |  |  | 2004 | 2006 |  | 2010 | 2012 | 2014 |  |  |  |  |  |  |  |
| 68 | 1936 | 2 |  |  |  |  |  |  |  |  |  |  | 2004 | 2006 |  | 2010 | 2012 | 2014 |  |  |  |  |  |  |
| 70 | 1934 | 2 |  |  |  |  |  |  |  |  |  |  |  | 2004 | 2006 |  | 2010 | 2012 | 2014 |  |  |  |  |  |
| 72 | 1932 | 2 |  |  |  |  |  |  |  |  |  |  |  |  | 2004 | 2006 |  | 2010 | 2012 | 2014 |  |  |  |  |
| 74 | 1930 | 1 |  |  |  |  |  |  |  |  |  |  |  |  |  | 2004 | 2006 |  | 2010 | 2012 | 2014 |  |  |  |
| 76 | 1928 | 1 |  |  |  |  |  |  |  |  |  |  |  |  |  |  | 2004 | 2006 |  | 2010 | 2012 | 2014 |  |  |
| 78 | 1926 | 1 |  |  |  |  |  |  |  |  |  |  |  |  |  |  |  | 2004 | 2006 |  | 2010 | 2012 | 2014 |  |
| 80 | 1924 | 1 |  |  |  |  |  |  |  |  |  |  |  |  |  |  |  |  | 2004 | 2006 |  | 2010 | 2012 | 2014 |
| 82 | 1922 | 0 |  |  |  |  |  |  |  |  |  |  |  |  |  |  |  |  |  | 2004 | 2006 |  | 2010 | 2012 |
| 84 | 1920 | 0 |  |  |  |  |  |  |  |  |  |  |  |  |  |  |  |  |  |  | 2004 | 2006 |  | 2010 |
| 86 | 1918 | 0 |  |  |  |  |  |  |  |  |  |  |  |  |  |  |  |  |  |  |  | 2004 | 2006 |  |
| 88 | 1916 | 0 |  |  |  |  |  |  |  |  |  |  |  |  |  |  |  |  |  |  |  |  | 2004 | 2006 |

*S2 Table: Longitudinal model predicting limitations in activities of daily living due to health reasons, full-succession of models*

|  |  | Model succession | | | | | | | | | | |  | Gender effects | | | | |  | Checks for selectivity | | | | |
| --- | --- | --- | --- | --- | --- | --- | --- | --- | --- | --- | --- | --- | --- | --- | --- | --- | --- | --- | --- | --- | --- | --- | --- | --- |
|  |  | Model 1a and 1b | |  | Model 2a and 2b | |  | Model 3 (pooled) | |  | Model 3  (pooled + recoded) | |  | Model 4a  (only women) | |  | Model 4b  (only men) | |  | Model 5a  (without controls) | |  | Model 5b  (SEM+FIML) | |
|  |  | England | Germany |  | England | Germany |  | Ref.: England | Difference of Germany |  | Ref.: Germany | Difference of England |  | Ref.: England | Difference of Germany |  | Ref.: England | Difference of Germany |  | Ref.: England | Difference of Germany |  | Ref.: England | Difference of Germany |
| Level at age 50 |  | 0.20*** | 0.07*** |  | 0.35*** | 0.15*** |  | 0.30*** | 0.60** |  | 0.18*** | 1.67** |  | 0.30*** | 0.57* |  | 0.30*** | 0.68 |  | 0.38*** | 0.55** |  | 0.35*** | 0.49* |
|  |  | (0.01) | (0.01) |  | (0.03) | (0.03) |  | (0.02) | (0.11) |  | (0.03) | (0.31) |  | (0.03) | (0.13) |  | (0.03) | (0.22) |  | (0.02) | (0.10) |  | (0.04) | (0.18) |
| Age/20 |  | 0.83* | 2.77*** |  | 0.69*** | 3.37*** |  | 0.72** | 4.13*** |  | 2.96*** | 0.24*** |  | 0.81 | 4.85*** |  | 0.61** | 2.91* |  | 0.67*** | 4.05*** |  | 0.79 | 6.98*** |
|  |  | (0.07) | (0.64) |  | (0.07) | (1.02) |  | (0.07) | (1.18) |  | (0.79) | (0.07) |  | (0.11) | (1.77) |  | (0.10) | (1.45) |  | (0.07) | (1.16) |  | (0.16) | (3.76) |
| (Age/20)² |  | 2.05*** | 0.80 |  | 2.03*** | 0.75* |  | 2.08*** | 0.35*** |  | 0.74* | 2.82*** |  | 1.97*** | 0.36*** |  | 2.23*** | 0.31*** |  | 2.11*** | 0.33*** |  | 2.00*** | 0.28*** |
|  |  | (0.14) | (0.11) |  | (0.14) | (0.11) |  | (0.15) | (0.05) |  | (0.09) | (0.41) |  | (0.18) | (0.07) |  | (0.26) | (0.07) |  | (0.15) | (0.05) |  | (0.27) | (0.08) |
| Cohort |  | 1.22*** | 0.74*** |  | 1.10 | 0.54*** |  | 1.20*** | 0.43*** |  | 0.51*** | 2.34*** |  | 1.13* | 0.42*** |  | 1.32*** | 0.45*** |  | 1.08 | 0.45*** |  | 1.08 | 0.33*** |
|  |  | (0.06) | (0.07) |  | (0.06) | (0.07) |  | (0.06) | (0.05) |  | (0.06) | (0.30) |  | (0.07) | (0.07) |  | (0.10) | (0.11) |  | (0.05) | (0.06) |  | (0.10) | (0.07) |
| Cohort#Age/20 |  | 0.83*** | 1.29*** |  | 0.86*** | 1.38*** |  | 0.82*** | 1.82*** |  | 1.49*** | 0.55*** |  | 0.84*** | 1.75*** |  | 0.77*** | 2.09*** |  | 0.88*** | 1.88*** |  | 0.85* | 2.12*** |
|  |  | (0.03) | (0.08) |  | (0.03) | (0.12) |  | (0.03) | (0.16) |  | (0.12) | (0.05) |  | (0.04) | (0.19) |  | (0.05) | (0.36) |  | (0.03) | (0.16) |  | (0.06) | (0.32) |
| Middle education |  |  |  |  | 0.53*** | 0.54** |  | 0.52*** | 0.92 |  | 0.48*** | 1.09 |  | 0.51*** | 0.84 |  | 0.57*** | 0.87 |  | 0.48*** | 0.94 |  | 0.48*** | 1.05 |
| (Ref: low) |  |  |  |  | (0.05) | (0.11) |  | (0.05) | (0.20) |  | (0.10) | (0.24) |  | (0.06) | (0.24) |  | (0.08) | (0.31) |  | (0.04) | (0.21) |  | (0.07) | (0.42) |
| High education |  |  |  |  | 0.16*** | 0.23*** |  | 0.16*** | 1.32 |  | 0.21*** | 0.76 |  | 0.17*** | 1.46 |  | 0.14*** | 1.27 |  | 0.16*** | 1.21 |  | 0.15*** | 1.42 |
|  |  |  |  |  | (0.03) | (0.06) |  | (0.03) | (0.44) |  | (0.06) | (0.25) |  | (0.04) | (0.65) |  | (0.04) | (0.65) |  | (0.03) | (0.40) |  | (0.05) | (0.32) |
| Middle education# |  |  |  |  | 1.25* | 0.84 |  | 1.28* | 0.72 |  | 0.91 | 1.40 |  | 1.29 | 0.71 |  | 1.21 | 1.09 |  | 1.15 | 0.78 |  | 1.15 | 0.58 |
| Age/20 |  |  |  |  | (0.13) | (0.20) |  | (0.14) | (0.17) |  | (0.20) | (0.34) |  | (0.18) | (0.21) |  | (0.21) | (0.50) |  | (0.12) | (0.19) |  | (0.22) | (0.25) |
| High education# |  |  |  |  | 1.63* | 0.81 |  | 1.67* | 0.50* |  | 0.83 | 2.02* |  | 1.90* | 0.29* |  | 1.54 | 0.93 |  | 1.39 | 0.59 |  | 1.67 | 0.37 |
| Age/20 |  |  |  |  | (0.33) | (0.25) |  | (0.34) | (0.17) |  | (0.24) | (0.71) |  | (0.53) | (0.14) |  | (0.47) | (0.54) |  | (0.28) | (0.21) |  | (0.58) | (0.21) |
| Middle education# |  |  |  |  | 1.06 | 1.35* |  | 1.06 | 1.29* |  | 1.37** | 0.78* |  | 1.16* | 1.24 |  | 0.94 | 1.24 |  | 1.09 | 1.28 |  | 1.12 | 1.45 |
| Cohort |  |  |  |  | (0.05) | (0.16) |  | (0.05) | (0.16) |  | (0.16) | (0.10) |  | (0.07) | (0.20) |  | (0.07) | (0.30) |  | (0.05) | (0.16) |  | (0.10) | (0.32) |
| High education# |  |  |  |  | 1.37** | 1.37 |  | 1.36** | 1.04 |  | 1.41* | 0.96 |  | 1.57*** | 1.03 |  | 1.21 | 0.91 |  | 1.37*** | 1.08 |  | 1.40 | 1.42 |
| Cohort |  |  |  |  | (0.13) | (0.23) |  | (0.13) | (0.20) |  | (0.23) | (0.18) |  | (0.21) | (0.28) |  | (0.17) | (0.28) |  | (0.13) | (0.20) |  | (0.30) | (0.84) |
| Middle education# |  |  |  |  | 0.98 | 0.95 |  | 0.98 | 0.96 |  | 0.94 | 1.04 |  | 0.93 | 1.00 |  | 1.06 | 0.86 |  | 0.99 | 0.94 |  | 0.98 | 0.92 |
| Age/20#Cohort |  |  |  |  | (0.03) | (0.07) |  | (0.03) | (0.08) |  | (0.07) | (0.09) |  | (0.04) | (0.10) |  | (0.05) | (0.14) |  | (0.03) | (0.08) |  | (0.05) | (0.13) |
| High education# |  |  |  |  | 0.93 | 1.05 |  | 0.93 | 1.10 |  | 1.03 | 0.91 |  | 0.83* | 1.23 |  | 1.04 | 0.99 |  | 0.96 | 1.03 |  | 0.91 | 1.07 |
| Age/20#Cohort |  |  |  |  | (0.06) | (0.11) |  | (0.06) | (0.13) |  | (0.11) | (0.11) |  | (0.07) | (0.21) |  | (0.10) | (0.20) |  | (0.06) | (0.13) |  | (0.11) | (0.22) |
| Refresh. Sample |  | 0.86* | 1.38*** |  | 0.90 | 1.31*** |  | 1.08 | |  | 1.08 | |  | 1.20** | |  | 0.91 | |  |  | |  | 0.99 | |
|  |  | (0.05) | (0.09) |  | (0.06) | (0.09) |  | (0.05) | |  | (0.05) | |  | (0.07) | |  | (0.06) | |  |  | |  | (0.07) | |
| Eastern part if Germany |  |  | 1.16* |  |  | 1.25*** |  | 1.22** | |  | 1.22** | |  | 1.13 | |  | 1.38*** | |  |  | |  | 1.21* | |
|  |  |  | (0.07) |  |  | (0.08) |  | (0.08) | |  | (0.08) | |  | (0.10) | |  | (0.13) | |  |  | |  | (0.11) | |
| Proxy Int. |  | 2.35*** | 4.82*** |  | 2.31*** | 4.58*** |  | 2.53*** | |  | 2.53*** | |  | 2.61*** | |  | 2.48*** | |  |  | |  | 1.00 | |
|  |  | (0.06) | (0.31) |  | (0.06) | (0.29) |  | (0.06) | |  | (0.06) | |  | (0.09) | |  | (0.10) | |  |  | |  | (0.00) | |
| Part. Selective |  | 1.43*** | 1.68*** |  | 1.38*** | 1.59*** |  | 1.36*** | |  | 1.36*** | |  | 1.41*** | |  | 1.29*** | |  |  | |  | 1.42*** | |
|  |  | (0.04) | (0.13) |  | (0.04) | (0.13) |  | (0.04) | |  | (0.04) | |  | (0.05) | |  | (0.05) | |  |  | |  | (0.06) | |
| Part. 1time |  | 1.35** | 1.51*** |  | 1.21 | 1.45*** |  | 1.34*** | |  | 1.34*** | |  | 1.53*** | |  | 1.09 | |  |  | |  | 1.54*** | |
|  |  | (0.15) | (0.10) |  | (0.13) | (0.10) |  | (0.07) | |  | (0.07) | |  | (0.11) | |  | (0.09) | |  |  | |  | (0.13) | |
| Gender (ref. male) |  | 1.16*** | 1.13* |  | 1.10*** | 1.02 |  | 1.09*** | |  | 1.09*** | |  |  | |  |  | |  |  | |  | 1.05 | |
|  |  | (0.03) | (0.06) |  | (0.03) | (0.06) |  | (0.03) | |  | (0.03) | |  |  | |  |  | |  |  | |  | (0.04) | |
| N of individuals |  | 11352 | 5573 |  | 11352 | 5573 |  | 16925 | |  | 16925 | |  | 9075 | |  | 7850 | |  | 16925 | |  | 16925 | |
| N of observations |  | 37431 | 10895 |  | 37431 | 10895 |  | 48326 | |  | 48326 | |  | 26112 | |  | 22214 | |  | 48326 | |  |  | |
| χ² |  | 4258.86 | 1985.16 |  | 4367.18 | 2000.58 |  | 6410.43 | |  | 6410.43 | |  | 3854.43 | |  | 2594.92 | |  | 4400.19 | |  |  | |
| Degrees of freedom |  | 9 | 10 |  | 17 | 18 |  | 31 | |  | 31 | |  | 30 | |  | 30 | |  | 25 | |  | 31 | |

* p<0.05, ** p<0.01, *** p<0.001; Displayed are incidence ratio rates (IRR) with standard errors in parentheses. Cohort is coded from earlier-born to more recent-born cohorts.

*In depth description of the robustness of the findings to gender differences and panel attrition*

Overall, the general pattern of results was stable, although we could identify some specific trends. First, gender differences are minor, with overall patterns being fairly similar for men and women. Models 1a and 1b identified 15 percent higher risks for an (additional) ADL for women than for men in England and in Germany 13 percent, respectively (*IRR_E_*= 1.15***; *IRR_G_*= 1.13*). After controlling for educational differences over age and cohort, level differences remain significant only in England (see Model 2 a) and b): *IRR_E_*= 1.10***). Second, more detailed investigations into gender differences (models 4a and 4b) reveal that lower levels of education for women result in slightly less disadvantage at the age of 50, and then follow a more limited converging development over age than men. All other developments seem to follow the same patterns independently of gender.

To evaluate the influence of panel attrition on our models, we excluded all control variables (refreshment samples, proxy-interviews, and participation patterns; see S2 Table Model 5). This model, which assumingly is more biased, indicates the directions of a misfit. We find that Models 3 and 5a only differ slightly. Interestingly, the controlled Model 3 shows more pronounced educational differences and in particular more pronounced educationally stratified developments over age than the uncontrolled, assumingly more biased Model 5. This suggests that selection is based on education. Effects of other variables did not change. Furthermore, we translated the model into the SEM framework to apply the FIML approach to tackle panel attrition. Compared with Model 3, the resulting Model 5b shows again highly similar findings. Three smaller differences can be found: First, the SEM shows less accelerative development over age but stronger linear age effects for both countries. Second, cohort differences seem to be less pronounced, but different cohorts diverge more over age. Third, while the SEM shows equal or smaller parameters for educational level and development, it shows stronger differences in ADL by education over cohorts. Since FIML addresses bias due to selective participation patterns, these findings indicate that the current research estimates levels and developments of educational status rather accurately but underestimates educational differences between cohorts. Summing up, both approaches point to accuracy in educational level differences but highlight different selection patterns based on educational interactions: one with age development and one with cohort. Accordingly, our results appear to represent a conservative but robust picture of health developments for different educational groups which might be even stronger in the entire population than in this sample.
