# Supplementary material for: Putting cumulative (dis)advantages in context: Comparing the role of educational inequality in later-life functional health trajectories in England and Germany
Source: PLoS One. 2020 Dec 30;15(12):e0244371. doi: 10.1371/journal.pone.0244371 (PMC7773250; doi:10.1371/journal.pone.0244371)
Supplement: Attachment — Submitted filename: Response_to_reviewers_0821.docx [file pone.0244371.s002.docx]

**Response to the reviewers for PLOSone manuscript: “Putting cumulative (dis)advantages in context: Later-life health trajectories in England and Germany.”**

Deadline: 27 Aug 2020.

**Comments of the editor:**

The reviewers raise a number of methodological queries and have requested further contextual information to develop the justification for the cross-country comparison. Please address each of the reviewers' comments carefully, either by making changes to the manuscript, or providing a clear explanation in the response to reviewers for why you have not made the changes suggested. I would like to add a couple of minor points to those raised by the reviewers.

1. One is to be careful **not to conflate** relatively **low levels of educational attainment** with **inequalities in educational attainment**. At the bottom of page 8, you base some of your hypotheses on greater educational inequality in England compared with Germany. However, in the background section you show that England had much lower levels of attainment than Germany, on average, in the 1970s; this doesn't necessarily equate to greater inequality. If England did indeed have greater levels of inequality, please provide that evidence in the background section.

- Response: Thank you for pointing out this ambiguity. Indeed, we conflated at some points in the manuscript “levels of” and “inequality in” educational attainment. We revised the theoretical background section quite strongly to be more precise in this regard and added some additional comparative statistics for both countries. To name only one example:

Page 9 f.: “As Table 2 shows, in 1950 a higher proportion of the population had completed upper secondary education in Germany compared to in England 20 years later, but there was always a slightly higher proportion completing post-secondary education in England.”

2. Also, you cite two studies that have previously investigated educational inequalities in functioning in these two countries (Jurges 2009 and Cambois et al 2009). Please make explicit in the text how your work builds and improves on this previous evidence.

- These two studies are cross-sectional in nature. However, they show nicely how health resources are unequally distributed over societies comparing also England and Germany. Therefore, we added them in Table 2 as sources to derive hypothesis based on their findings. As pointed out in page 12 f., the strength of our study is to compare education-differentiated developments of health over age and cohort – in a longitudinal fashion – which enables us to separate processes of ageing and differences between birth cohorts.

**Comments of the reviewer:**

**Reviewer #1:**

The paper has an interesting premise, but I find that there are some major conceptual issues that the authors should address.

1. A major question I would like the authors to address is whether we can we **really study cohort** effects with these data, and whether the analyses really disentangle age and cohort, as **the overlap between age and cohorts is very limited**. I think some of the results reported stem from extrapolating a lot across time, e.g. we do not know what ADLs were like at age 50 for the earlier-born cohorts, as they have not been observed at this age. **Period effects** also remain unaddressed.

- Response: Thank you for your thoughtful comments. As a general remark: The line of questions you have raised, made us realize that in the previous introduction we missed the opportunity to explain why we prioritize cohort differences and panel attrition as the key processes to understand when investigating CAD processes (defined as diverging health development over age by different educational classes) and country context. Accordingly, the reader might have wondered why cohort (and related constructs as period) received so little attention. We apologize for that.
- In the revised introduction we now clearly state that the central assumption of the paper is that CAD processes would profit if they are compared between different contexts. Accordingly, most central to the paper is education-specific development of functional health (CAD) and its comparison between England and Germany. To do high-quality analysis of CAD processes, previous research (e.g. Wilson et al. 2007) pointed out that researchers need to address three central tenets including cohort differences and panel attrition. We moved this paragraph at the end of the introduction to show that it is less central to the paper.
- The methodological approach here applied in the paper follows closely the state-of-the-art approaches (e.g., Mirowsky & Kim, 2007 in Sociological Methods and Research, see new Table 1 – “Cohort-sensitive CAD studies”). Having not all information for all cohorts (e.g., health status for earlier-born cohorts at the age of 50) is a general limitation of survey-based analyses. An advantage of the current approach is to reveal that each cohort might follow a different development – and also to openly show for which cohorts information is available (see Figure 1). Based on the underlying cohort succession, these models allows us to show the general trend of cohort-sensitive developments.
- Finally, period cannot be addressed sufficiently in the paper because the paper focusses on age development and tackles already cohort differences into account. Cohort, age and period are co-dependent, accordingly only two effects can statistically be partialized out. Nevertheless, we address this weakness now in the limitation section stating:

Page 29: “Due to the main focus of the paper of the interaction of health development, educational class and country comparison, we acknowledged cohort-effects only as a linear trend. […] In a similar vein, period effects addressing for instance economic changes are out of scope of the current paper.”

2. The modelling assumes that there is a linear trend in the outcome by successive cohorts, for which there is no strong justification in the text. Different cohorts may have differences between them because of factors related to e.g. specific conditions such as war and famine that the cohorts experienced in a sensitive age. The authors have **not tried to check to what extent a linear trend across cohorts** would be the best way to conceptualise cohort differences by comparing the cohorts by categorising this variable. I would recommend that the authors justify how they treat the cohort variable based on both **theoretical grounds as well as by checking the data**.

- When we wrote the manuscript, we discussed this issue. We came to the conclusion that we need to focus on the non-linearity over age (due to the acceleration of functional limitations in later life). The development of health over age is more central to the paper than the succession of cohorts, which – as we point out now more clearly in the introduction – is an important aspect in life course studies but which can not be tackled in the same depth. The reason for this lies both in theoretical and in methodological limitations: In particular in a paper which also deals with processes of cumulative social inequality in two country contexts, it is very difficult to theoretically deduce hypothesis in which direction earlier-born cohorts additional differ from an overall cohort main trend. Moreover, we use interaction-effects to quantify and test differences between both countries in the central variables. While applying statistical testing of country differences is a central strength of the paper, we already apply three-way interactions, which are already difficult to interpret. Adding non-linear cohort-differences would further increase the complexity.
  In descriptive analysis, we did at the beginning of our project, we found no hint for cohort particularities. For instance, the mean levels by cohort for the full data set presented in Table R1 shows the expected intertwined pattern of age and cohort in which in particular higher ages show increasingly higher levels of ADL.

*Figure R1) Mean levels of ADLs over Cohorts by country*

|  |  | Cohort |  |  |  |  |  |
| --- | --- | --- | --- | --- | --- | --- | --- |
|  |  | 0. 1955/64 | 1. 1947/54 | 2. 1939/46 | 3. 1931/38 | 4. 1923/30 | 5. 1914/22 |
| England | mean | 0.22 | 0.23 | 0.29 | 0.43 | 0.63 | 0.88 |
|  | sd | 0.73 | 0.74 | 0.80 | 0.97 | 1.12 | 1.29 |
|  | N | 2,871 | 10,353 | 9,981 | 7,962 | 4,712 | 1,554 |
| Germany | mean | 0.12 | 0.10 | 0.16 | 0.28 | 0.52 | 0.87 |
|  | sd | 0.53 | 0.44 | 0.60 | 0.83 | 1.11 | 1.32 |
|  | N | 2,272 | 3,146 | 2,683 | 1,845 | 796 | 155 |

- While some research fields, in particular demographic and epidemiologic research, deal with the question if later-born cohorts are healthier than earlier-born cohorts and therefore might be more interested in a more advanced modeling of cohort-developments, the current paper is has set it’s focus stronger on educational differences. Similar to comment #1 on period effects, we this shortage in the limitations section expanding a sentence already pointing to this issue:

Page 29: “*Due to the main focus of the paper of the interaction of health development, educational class and country comparison,* we acknowledged cohort-effects only as a linear trend. However, cohort changes in health might be curvilinear or cohort-specific in response to critical historical events.”

3. Why did the authors include **age and age2** in the models? I didn’t see any justification for this or whether other polynomials were tested.

- We reply to this question together with the following comment.

4. Why do the **interactions with age only interact with age and not age2**? It is very difficult to interpret the interactions because they seem to ignore the non-linearity in the age effects that has been added in the models. I am not sure if the interactions have therefore been interpreted correctly. I think the **conclusions should be drawn from what the figures show**, rather than the statistical significance of interaction terms. For example, on page 25 line 3 it is stated that there is a higher acceleration in ADLs in Germany than England, but it doesn’t look that way from Figure 1.

- As mentioned above, development of health over age is central in the paper. Accordingly, we are grateful for pointing out in comment #3 this vacancy out. We added a following sentences in the “Separating age and cohort effects” paragraph in which we now make the point that (depending on the health facet under scope) the development does not follow a monotonous pattern but may show an accelerating increase over age which we needed to address using age².
- However, understanding the interaction of age and education to reveal processes of CAD needs only to take into account the mean-trend of age. While the age²-parameter indicates non-linear deviation from the mean-trend over the later life, our focus of the study lies in the question whether or not differences between educational classes increase systematically over the age 50 to 90. We did not raise the question if educational differences increase with increasing age faster or slower than on average (what the age²-interaction would indicate). This question would have needed much theoretical thinking presumably based on ambiguous trends. Also, in regard to the following comment #4, a strength of the applied analytic approach is that we can quantify differences (between educational groups or countries) using statistical testing. Relying on visual interpretations can be misleading, in particular if case numbers become small. This is the case for the mean-level acceleration of Germans ADLs-development: Although the non-linear effect of age² is smaller than in England and less easy to identify in Figure 2, the model parameter is statistically significant (as shown in Model 2b).

5. Could the fact that there an individual can have a **maximum of 5 ADLs** create a ceiling effect that may also explain any potential reduction in educational differences by age? E.g. by age the number of ADLs increase in all educational groups, but because this variable is restricted to a maximum 5, it means that the **highest educated would catch up with the lowest educated in later age**.

- We did also wonder whether or not our results were a statistical artefact based on the measurement of functional health. In particular because only very few observations with more than 4 (1.30 %) and more than 5 ADLs (0.78 %) – and as expecting these were found mostly in the highest age groups and for the lower educated (in both countries).
- We estimated the model also as binary-logistic regression with compares having no limitation with having 1+ ADLs. In contrast to poison-distributed regression in which each value of the dependent variable affects the mean-level estimates of the trajectory, binary-logistic approaches are based on a (more or less arbitrary) threshold which might affects the mean-level findings. However, comparing these both models shows that despite the level-differences, the education-specific trends do not differ. Accordingly, we would argue that the results are robust to the coding of the variable.

*Binary logistic regression	Poison-regression*

|  |  |
| --- | --- |

Minor issues:
6. p.14 line 1-2 Is the wording here correct? “increase in one unit in the dependent variable” or “independent” variable?

- We actually meant to use two times “dependent variable” but the sentences was very difficult to understand thus we revised it.

  old:
  These models are very similar to linear growth curve models (with random intercepts and fixed slopes), except that they allow an increase of one unit in the dependent variable to have a variable effect over the range of the same dependent variable.

  new:
  These models are very similar to linear growth curve models (with random intercepts and fixed slopes), except that an increase of one unit in the dependent variable does not linearly depend on an effect of an independent variable but can vary over its range.

7. Why was participation rate in 2010 so low in the German SHARE data?

- This is a good question. The methods report on participation rates shows no particular changes in the retention rates for wave 4, however, wave 4 is the only wave included which received no refreshment sample, which might explain the lower case numbers compared to the other waves.

Source: Bergmann, M. Kneip, T., De Luca, G., & Scherpenzeel, A. (2019). Survey participation in the Survey of Health, Ageing and Retirement in Europe (SHARE), Wave 1-7. Based on Release 7.0.0. SHARE Working Paper Series 41-2019. Munich: SHARE-ERIC.

8. There are some contradictory sentences in the discussion, such as when on page 22 the authors state that prior CAD research hasn’t found reducing inequalities with age, but then on page 25 discusses an additional two studies (references 9 & 55) that have found that CAD is not a "later-life process". To my knowledge, it is commonly found that there is some reduction in health inequalities in later life to a large part due to survivor bias, which I have not seen the authors discussing.

- Thank you for pointing this out. It seems that this sentence was misleading. We wanted to point out that CAD process also take place in earlier life phases to provide an explanation for the level differences by education in England (and smaller also for in Germany) at the age of 50. We revised the sentence.

9. Unclear sentences/proof reading needed: p.9 line 1-2, p.23 lines 20-21, p. 25 lines 5-7

- Thank you for your detailed feedback! We rewrote these sentences to be more precise and also proof-read the entire article again.

**Reviewer #2:**

This study aims to compare the educational gradient in functional health development among older people in England and Germany, using longitudinal data. Although this paper is interesting, the authors should strengthen the rationale and the empirical analyses before publication. Below are some questions and comments that I hope the authors find useful.

1. While the introduction reads well, as a reader I would have preferred the **focus on the country context**. My understanding is that the main contribution of this paper is to further the research on CAD processes in two European countries. I would therefore encourage the authors to better describe what is **known about these two countries, their educational levels, and their health**. For instance, you mention that in Europe the educational health gap is smaller than in the US but it would be useful to provide more details about this. Also, what have those few comparative studies found so far? And what does this study add to the existing knowledge?

- Response: We followed your advice and entirely rewrote the introduction focusing stronger on the country context. In this process, we strengthened the comparison of England and Germany using further indicators to describe differences in the institutional arrangements. This also helped to better address the ratio behind choosing England and Germany as important cases (see your comment #2) and also to deduce hypothesis for country comparison (comment #3).

2. The **choice of Germany and England should be better justified**. Do these countries offer sharp or nuanced contrasts with regard to the social conditions and institutional factors which may shape individual health trajectories and their associations with education? Also, given the history of Germany (with long-standing East/West divide) I would expect a better description of those policies likely to affect both the health profile and the educational gradient. Not sure whether you would have enough power, but one could also consider to analyse East and West Germany separately.

- Thank you for pointing out this shortage. In the revised introduction, we aimed to be clearer on this topic arguing that the comparison is based on an analytical interest to compare the associations of education-age-health in two contexts which differ in the distribution of education, differ in the link of education on health and are rather similar in the accessibility of the health care system (page 7 ff.). We explain each point with macro indicators and deduce assumptions how this might affect the CAD processes.
- Finally, regarding to the question of the two German states: While this might have been an interesting question to study on its own, more than controlling for potential effects as we did would lay out of the scope of the paper – and as suggested – would lead to serious statistical problems based on case numbers (in three educational classes over 7 age categories). Moreover, data set at hand started in 2002, this is 12 years after reunification and also 12 years of joint institutional regulations. However, regional differences between the two former parts still exists, e.g. in economic strength (both on individual and federal level), family norms and access to elites. Accordingly, the paper would need to focus more strongly on this to disentangle CADH processes from early-life experiences. This might be an interesting paper to write/read.

3. A better description of the countries would also help the readers understand where your hypotheses come from. For instance, I was unsure why you hypothesise that the health profile of Germany and England is similar, given that studies which have compared SHARE and ELSA tend to suggest that age-standardised limitation in one or more ADL or IADL tends to be lower in SHARE than ELSA.

- In the revised introduction, we also attributed to this comment. We think we found the article you were referring to (Cambois et al., 2009 [55]). We added this information in Table 2, which aims to provide an overview for comparing the countries using macro-level indicators. As you recalled, these numbers point out that while the health-inequality (indicated by ADLs) seem to be on a similar level for England (high to low educated: 19 %-points for 50-64 years) and Germany (23 %-points). Nevertheless, Germany shows higher average-levels for ADLs (41% for middle educated age 50-64 vs. 25%). Accordingly, we revised our argumentation with a focus on being more precise on level differences and social inequality differences.

4. You mention that some country and educational differences might depend on the health outcomes used. So, **why did you consider only ADLs**? This needs a better and more articulated justification than the one sentence on page 8 and the brief rationale on p11. Also, given that previous comparative studies have considered a range of different health measures, I wonder whether it would be worth considering other measures too to test if similar educational trajectories would hold also for chronic diseases or long-standing illnesses for instance.

- When revising the introduction, we also updated the literature review. Since many new studies have been published since the first draft of this paper was written, we decided to less focus on the landmark study of Wilson et al. from 2007 and to give a systematic overview of research in different fields of health development and CAD have been published (Table 1). In this context, we also present systematically which health dimensions the different papers have been addressing to reveal that it is important to be precise in terms of health dimension in the field of health research. The scope of the current paper lies on the intersection of health development over age for different educational groups in two distinct although similar countries. ADLs are best suited to be compared between countries. Adding more health dimensions would excide this scope – however, we are positive that this scope is now better embedded in the frame of the paper.

5. Finally, I assume that the two countries also differ in the extent of educational disparities in smoking, obesity, and physical exercise (most likely with larger differences observed in England). If such cross-country educational disparities in health behaviours do exist, I wonder whether it is worth both mentioning them and also controlling for these factors in your analyses?

- Thank you for suggesting further underlying “behavioral” mechanisms for differences in education-stratified health. This might be an additional country comparison argument to those stated from us on the bottom of page 8. The scope of the study is to reveal that the stratification in health differs over the life course between England and Germany. Future research might want to focus stronger on the underlying mechanisms, for which indeed health behavior might play an important role, next to institutional differences.

6. There are some methodological decisions which are currently not well described in the paper and which do not seem to be convincing
a. Why were partners excluded whereas proxy respondents were not? Did you exclude only younger partners or all partners even if they were ‘core members’ of the study? The target population of SHARE is all individuals aged 50 and older and their partners, independent of age.

- The reason behind this selection is that both studies (SHARE and ELSA) focus in their sampling procedure on the main respondent. Accordingly, they allow also only for these respondents to provide representation of the sample population. When including partners more cases are added but since these are not part of the sampling framework and they are also not independent from the main respondents, they would bias the informative value of the study population.

b. Why did you exclude the refresher sample of ELSA in Wave 4? The IFS provide education for all sample respondents (via UK data service). Also, substituting missing values with their partner’s level of education should be better justified given gender differences in educational attainments among older people. Also, if you exclude Wave 3 for SHARE, should you not also exclude Wave 4 of ELSA to maintain comparability?

- Major parts of the harmonization of both datasets are based on the code provided by the Gateway to global ageing project (g2aging.org). This is a project is specialized in harmonizing a multitude of aging studies. Applying their code reduces the coding procedure significantly and reduces further the risk of coding errors.

Regarding the question of excluding the refreshment sample of wave 4, we used the by the g2aging project provided ISCED-variable. While this variable is part of the distribution of SHARE, in the ELSA a less international-comparable education indicator is provided (edqual – variable based on NVQ). These two indicators can not easily translated into each other (see Schneider: The application of the ISCED‐97 to the UK’s educational qualifications available under <http://www.mzes.uni-mannheim.de/publications/misc/isced_97/schn08e_the_application_of_the_isced-97_to_the_uks_educat.pdf>), additional information seems to be necessary. While in the g2aging documentation it is not specified which information they rely on, they state in their data manual that in the HSE this information was for the refreshment sample w4 not available (see also Table R2). “A special missing code .h is used when the respondent is missing a value for educational qualifications because the respondent reported their educational qualifications during an HSE survey of which the values have not been released by ELSA” (G2Aging project - Data Manual Version E). As background information: The refreshment samples of ELSA are based on the Health Survey for England (HSE) and for wave 4 on the HSE survey of 2006. Accordingly, we excluded the refreshment sample wave 4 and explained this on page 16.

*Table R2: Education by sampling pattern in ELSA*

```
                      |                             raeducl:R harmonized education
             cohort_e | 1.less th  2.upper s  3.tertiar      .d:DK  .h:missin  .m:Missin   .o:other  .r:Refuse |     Total
----------------------+----------------------------------------------------------------------------------------+----------
1.original HSE sample |     4,945      4,298      1,274          9          0         81        985          1 |    11,593
2.partners of origina |        80         90         34          3          0          8          4          1 |       220
3.young partner of or |       117        312         95          0          0         10         21          2 |       557
4.w3 refreshment samp |       308        652        317          1          1          1          5          1 |     1,286
5.partners of w3 refr |        16         26         20          2          0          0          0          0 |        64
6.young partner of w3 |        57        176         73          0          0          0          0          1 |       307
7.older partner of w3 |        56         65         34          1          0          0          0          1 |       157
8.w4 refreshment samp |       293        247        106          7      1,664          0          0         11 |     2,328
9.partners of w4 refr |        16         23          6          0          0          0          0          0 |        45
10.young partner of w |        28         38         14          0         42          0          0          1 |       123
11.older partner of w |        33         30         16          3         87          0          0          1 |       170
12.w6 refreshment sam |       160        386         47          2          0        262          0          2 |       859
13.partners of w6 ref |         4          7          7          0          0          0          0          0 |        18
14.young partner of w |        22         67         11          2          0         47          0          0 |       149
15.older partner of w |        45         46         11          0          0         46          0          1 |       149
16.w7 refreshment sam |        54         94         14          4          0        136          0          2 |       304
17.partners of w7 ref |         0          2          0          0          0          0          0          0 |         2
18.young partner of w |         7         27          1          0          0         38          0          0 |        73
19.older partner of w |        16         23          4          1          0         31          0          2 |        77
----------------------+----------------------------------------------------------------------------------------+----------
                Total |     6,257      6,609      2,084         35      1,794        660      1,015         27 |    18,481
```

- Regarding the second comment: The argument behind using the partners educational level was that excluding 8 % of the sample with missing information on education would result in a systematic downsize of the sample. As a remedy for this potential bias, the best information available is partner education status (for 5.4 % of the ELSA study). Studies on partner selection have a long-standing tradition to show that partners more often share a joint educational background (status homogamy). We understand that women in higher ages tend to have in particular lower educational levels, leading to more often falsely classified higher-educated men of earlier-born cohorts into the lower-level educational classes. While this seems to be a valid problematic point, the impact on the study might nevertheless low since only 5.4 % of the study population showed missing and this problem affects thereof only men of the earlier-born cohorts. Therefore, we attribute the concern with an explaining sentence in the sample selection paragraph:

Page 16: “Based on studies showing that partners tend to share similar levels of education (Kalmijn 1990), if possible, we substituted missing information on education with the partner’s education level (5.4 %). This approach reduces systematic drop-out although it might lead to minor misclassifications: For instance, women did profit stronger from the educational expansions which results in that in particular men of earlier-born cohorts might be receive lower levels of education.”

- Finally, we actually excluded the complementary wave of SHARE-life in the ELSA study (wave 3). This was mentioned in the Data paragraph: “We only used waves common between both studies (2004 to 2014, excluding 2008, as the health items were not part of SHARE that wave, see Table 2).”

c. Germany is known to have a very high attrition rate between waves. Could you specify how many of those 5,500 respondents were interviewed at all waves? A similar description would be useful for ELSA too. At the moment, it is unclear who the respondents are and what proportion was only interviewed at only 1 wave. Also, I wonder whether it would be worth presenting Table 1 restricting the sample to those present at all waves?

- Germany had in particular a low retention rate for the first re-participation (w1 🡪 w2) with ~55 % and for the second to the third wave (58%). For the following waves, including the refreshment samples, attrition rates were fairly average (with above 70%; see Bergmann et al: <http://www.share-project.org/uploads/tx_sharepublications/WP_Series_31_2017_BergmannKneip_01.pdf>) which seem to be on average on the same level as the ELSA study shows (ELSA cohort profile <https://academic.oup.com/ije/article/42/6/1640/735886> )
- Additionally, we analyzed the participation patterns of our analysis sample:

*Table R3: Participation patterns in the ELSA and SHARE (Germany) study*

- England:

```
     Freq.  Percent    Cum. |  Pattern*
 ---------------------------+----------
     4008     35.31   35.31 |  11.111
     1452     12.79   48.10 |  11....
     1225     10.79   58.89 |  1.....
      857      7.55   66.44 |  .1.111
      689      6.07   72.51 |  11.11.
      580      5.11   77.62 |  ....11
      537      4.73   82.35 |  11.1..
      422      3.72   86.06 |  .1....
      226      1.99   88.05 |  .....1
     1356     11.95  100.00 | (other patterns)
 ---------------------------+----------
    11352    100.00         |  XX.XXX
```

- 35% continuous participation
- Low proportion of refreshment: max. 5.1%
- Germany:

```
     Freq.  Percent    Cum. |  Pattern
 ---------------------------+---------
     2093     37.56   37.56 |  ....11
      884     15.86   53.42 |  ....1.
      855     15.34   68.76 |  1.....
      427      7.66   76.42 |  11....
      322      5.78   82.20 |  11.111
      312      5.60   87.80 |  .1....
      189      3.39   91.19 |  11.1..
      180      3.23   94.42 |  .1.111
       90      1.61   96.03 |  .1.1..
      221      3.97  100.00 | (other patterns)
 ---------------------------+---------
     5573    100.00         |  XX.XXX
```

- 6.8 % of our sample participated at each wave,
- 15.3 % only at the first wave (fits to low retention rates at w1-w2)
- Major parts of our sample was part of an refreshment sample starting in wave 5 with follow up (37.5 %) or without (15.9 %)
- Differences in participation patterns do not only emerge between different studies but also between different life phases, regions and interests in the topic of the study. To deal with the different participation patterns, we included a variable representing a 1-time, several times and continuous participation in the models.

d. If ELSA had refresher samples in Wave 7 (the latest one you consider), how is it possible that only 1% of your final analytical sample was a one-time off (see p.13)?

- The refreshment sample of ELSA wave 7 is coded as “complete participation”, since it had only one-time chance to participate. Generally, refreshment samples for England were included at wave 3, 6 and 7 (we used only ELSA waves 2-7). As shown in the response to comment #6c, in England only a small group of individuals participated one-time only. This might be based on the fact that the first wave was excluded in ELSA which typically shows the lowest retention rate. The 2 % who participated only in wave 7 can belong to the group who fully participated if they were respondents of the refreshment sample wave 7 or one-time only if the belong to refreshment sample wave 6. Please note that the indicator is already generated before the analytical sample is selected (e.g., excluding wave 4). Therewith, it represents the respondent’s participation pattern in the ELSA study and not only in he applied subsample.

e. Why did you not consider using the toilet as one of the ADL difficulties? I might be wrong but I think both surveys include this item.

- Indeed, this item is available in both data sets as part of the ADL assessment. We were aware of this item and run both analysis with a 5-point and a 6-point ADL-score. The patterns didn’t differ therefore we decided to use the from g4aging provided 5-item version since it is easier comparable to the HRS and many other major studies worldwide. We added this information as a footnote.

f. It appears that you have already conducted analyses separately by gender. Given that gender seems to be significant in all models and that other studies have found stronger increase in health gaps between education groups among women, I would present the models separately.

- We did as part of the Appendix Table S2: Models were run separately for women and men. As you assume, we also found a stronger CAD-effect for women than for men. We could not elaborate on this in more detail but we saw that this might be of interest for some readers.

g. Can you specify how you categorise German Eastern and Western respondents? Is this based on their current residence or on where they were born?

- The information is based on the current region the respondent is living in. Accordingly, it represents health-differences between the population of “East” and “West”. While this did not account for the major migration between both German states which took place following 1990, it stronger addresses structural differences which currently exist, e.g., family formations, public transport coverage, social norms. Certainly, good arguments can be found for both indicators. We improved the detail on this information on page 17.

h. I would appreciate it if you could better explain Model 3. In particular, what does the ‘difference of Germany’ column in Table 3 represent? I am not sure I understood how to interpret those values.

- We revised the section on page 20 and hope that is now easier to understand.

7. The description of you results is at times difficult to follow. I understand that you have many tables and results to show. However, it would be best to focus on those findings which highlight the main contribution of your study and compare the educational gradient in the two countries under study. The section on robustness checks could be considerably reduced.

- Thank you for the suggestion. We revised the results section and reduced information on robustness checks to a minimum in the manuscript and added the detailed information into the supplement.

8. Why did you present in Fig 2 only three cohorts? Commenting in the main body on just a few of the trends is fine, but it would still be useful to present a figure with all cohorts under study.

- We did so to simplify the graph. In the original graph, for each country 21 lines (7 cohorts with 3 educational categories) prohibited, due to too much detail, to understand the bigger picture. Also, it is easier to see that the latest-born low-educated in England at age 55 have almost an equal level of limitations as the mid-educated of the mid-cohort, 20 years older in age. We made this ratio now clearer by adding the reason in the regarding sentence: “Fig 2 illustrates the results of Models 2a and 2b – for reasons of visibility – only for three exemplary cohorts (1947/54, 1931/38, 1914/22).” (pp. 24)

9. In your discussion, it would help readers if a clearer statement was made about what we learned from the study that we did not know before. In other words, what is the story or punch line here? **The authors should make an effort at pulling the results together in an integrated fashion.** Also, you should link/relate your own findings to the existing literature. How do your findings contradict previous studies? And what could be the potential reasons for such unique results for England?

- We now also revised the discussion section. In the updated version, we clearly state at the end of the first paragraph what our study contributes to the understanding of CAD processes in health. We discuss now stronger the study findings in the light of macro-level country indicators, newly introduced in the introduction, and cut down methodological arguments regarding panel attrition.

**Reviewer #3:**

I enjoyed reading this manuscript, but several issues should be addressed.

1. The manuscript rightly states that “complex, multi-dimensional construct [46], researchers need to be very clear about which specific dimension of health they analyse.” Therefore, the health outcome should be explicit in the **Title as well as throughout the manuscript**. This study focused on ADL limitations, and we don’t know whether the results in England and Germany would differ if another outcome was used, say cognition or blood pressure. Therefore, the **findings should not be overstated, especially in the Conclusion.**

- Response: Thank you for pointing this out! We already had this in mind as we introduced the health dimension of “functional health”. Nevertheless, we have been inconsistent in the first version of the manuscript and accordingly we revised the manuscript, better introducing the dimensions of health (also in the newly added Table 1 with an overview of previous research) and being more precise in the discussion.

2. The authors did a nice job with sensitivity analysis on attrition within each cohort, but it appears that **the attrition was worse in German SHARE than in ELSA**? Even after including the refreshment samples, the mean number of ADLs measures are 4.0 in ELSA and 2.5 in SHARE-Germany. The **response rates for each study should be reported in Methods**. If attrition was larger in one study than in another, this should also be **mentioned as a study limitation in the Discussion**. Even with careful methods to deal with attrition within a study, could this influence the differences in results found between England and Germany?

- We understand that the differences in the number of re-participations received not enough attention. We therefore added some information. The retention rates actually do not differ so much between England and Germany (except for the w1-w2 retention in Germany was quite low). The difference in average number of re-participation is stronger based on the survey design: ELSA applied a huge baseline sample with small refreshment samples while in SHARE Germany, a substantial number of cases steam from refreshment samples, in particular also from the last two waves.

*Table R4: Refreshment samples in ELSA and SHARE (Germany)*

```
England:

           |                         wave
   refresh |         1          2          4          5          6 |     Total
-----------+-------------------------------------------------------+----------
      No 0 |     8,482      7,285      6,084      5,491      4,741 |    32,083
     Yes 1 |         0      1,267        947      1,581      1,553 |     5,348
-----------+-------------------------------------------------------+----------
     Total |     8,482      8,552      7,031      7,072      6,294 |    37,431


Germany:

           |                         wave
   refresh |         1          2          4          5          6 |     Total
-----------+-------------------------------------------------------+----------
      No 0 |     1,964      1,038        631        498        392 |     4,523
     Yes 1 |         0        609        274      3,204      2,285 |     6,372
-----------+-------------------------------------------------------+----------
     Total |     1,964      1,647        905      3,702      2,677 |    10,895
```

- To tackle this, we added a sentence in the data paragraph:
  Page 17: “Note that the mean number of observations does differs between England (Ni = 4.0, SD = 1.3) and Germany (Ni = 2.5, SD = 1.3), but less because of major differences in retention rates but rather due to the relatively larger refreshment samples included in the later waves of SHARE Germany [81,82].”
- We still included these cases although they help not to differentiate between within and between variance, they provide information on between person differences and help therewith to reduce panel attrition bias of the previous samples. To reduce potential differences based on the survey design (but also for panel attrition), we included variables on the study participation (full, partly, 1-time only) and whether or not the respondent is part of a refreshment sample.

3. Abstract>Background: I would replace the word “development” with “decline,” since technically both baseline samples include people who have already developed functional limitations.

- We understand that the original version was misleading. We revised this section of the abstract entirely reading now:
  Page 4: “The cumulative (dis)advantage perspective more and more is examined in a comparative way, to highlight the role of context in generating inequality over the life course. This study adds to this field of research by examining trajectories of activities of daily living (ADL) in later life by educational level in a country comparison of England and Germany, emphasizing differing institutional conditions.”

4. The Background>Country context should be strengthened with a more critical review of the available evidence. Findings from previous studies are not very informative because the health outcomes in these studies are only reported for a few of them. If possible, more attention should be paid on the studies analysing objective health outcomes instead of self-rated health which can be problematic since the concept can change as people age due to changing expectations, as well as between cultures. If word count is an issue, the earlier sections can be cut down.

- Thank you for your advice. We revised the introduction with a stronger focus now on country context (using macro-level indicators for describing differences). We included also more information on different health dimension to underline stronger the strength of focusing on functional health. See also comment #5.

5. Is it really true that that CAD processes have generally been examined in single countries?
<http://dx.doi.org/10.1136/jech-2015-206548>
<https://doi.org/10.1017/S0144686X19001740>
The first paper looks at CAD processes in physical functioning in 3 European countries.
The 2nd paper doesn’t mention the hypothesis explicitly but shows differences in CAD processes between 4 countries.
These are only two examples, but available evidence should be reviewed appropriately and included in the paper as relevant. Depending on the available evidence, the authors should reconsider the claim that cross-country research in this area is largely absent.

- This manuscript took its time to get ready for submission. In that time, an astonishing amount of papers got published in that field – some also focusing on context differences. We therefore decided to change the story of the manuscript from the previous version featuring the flagship article of Wilson et al. (2007) to the current stream of publications on development and CAD in health. Therefore, we add a new Table X giving an oversight of this work and – related to comment #4 – show which health dimensions they have been addressing.

6. Methods>Page 10: It’s the English Longitudinal Study of Ageing, not **on** Ageing

- Thanks a lot!

7. Table 2: This should report medians and IQR since ADLs are a count. If ADL scores ranging from 0-5 and the means are all below 1, this shows the data are not normally distributed.

- Right, we were aware of this point in the regression models but not in the Descriptive information. Thank you for pointing this out. However, when we updated Table 2 (now Table 3) we found that for each cell the median = 0 and IQR = 0 or 1. Therefore, we stick to the previous version of the table (to give at least some information).

8. Results>page 18: The ELSA results IRR 0.12 and IRR 1.23 don’t match the IRRs reported in the Table. The pvalue for IRR 1.63 is below 0.05 in the Table but below 0.001 in the text. Please correct.

- Thank you for your thorough reading! We apologize for the mistakes. We have corrected them and double-checked all other numbers again.

9. Somewhere in the manuscript, it would be good to mention the larger public health significance for ADL limitations. Why is it important?

- We already had this included in the paragraph of policy implications on page 21:

“The functional dimension of health is more strongly related to the need for care, rehabilitation and medical assistance than, for instance, indicators of physical or emotional health [70].”

- To guide the reader stronger through the manuscript, we additionally mention this information already when we introduce the health dimension on bottom of page 11 f. and page 12.
