# Supplementary material for: Putting cumulative (dis)advantages in context: Comparing the role of educational inequality in later-life functional health trajectories in England and Germany
Source: PLoS One. 2020 Dec 30;15(12):e0244371. doi: 10.1371/journal.pone.0244371 (PMC7773250; doi:10.1371/journal.pone.0244371)
Supplement: Attachment — Submitted filename: Response_to_reviewers.docx [file pone.0244371.s003.docx]

**Response to the reviewers for PLOSone manuscript: “Putting cumulative (dis)advantages in context: Later-life health trajectories in England and Germany.”**

Deadline: 27 Aug 2020.

**Comments of the editor:**

Dear Martin,

You and your colleagues have done an excellent job of responding to review comments. Reviewer 1 continues to have some queries regarding the manuscript, some of which would be useful to address before publication.
> Response: Thank you very much. We did put much afford in the first round of R&R because of the many constructive comments we received. We are very happy that we were able to address most of them adequately.

R1 asks for an explanation regarding the sentence 'The 12-years age range is based on 10 years of observation plus 2 additional years one at the beginning and one at the end of the period which rely n the clustering of the age variable in 2-year brackets" on p. 18. I am equally unclear as to what you mean by this. Please could you explain?

> R: We revised this sentence to:
“The 12-year age range is based on a 11 years of observation period of the applied data (2004-2014) plus one year due to our categorisation of age groups. As shown in Supplement Table S1, a person born in 1948 is 56 years old at the first observation in 2004. He or she can be traced until age 66 in 2014. Due to the clustering of age in 2-years intervals, a person born one year later would be included in the same health trajectory (age 57 to 67).” (p. 18)

R1 also mentions formatting changes to Fig 1 and adding a legend to Fig 2 which I agree would help the readers' understanding of these figures.

> R: These were very good suggestions which we were happy to adapt.

In their original comments on the finding of reducing inequality in older ages, R1 raised the issue of survivor bias. You have not commented on this in your reply and have not added it to your Discussion text as a potential explanation for these age patterns. Please could you add this or explain why you feel it is not appropriate to add this?

> R: Unfortunately, we missed the chance to discuss this comment in the last R&R although the idea is very helpful to explain our findings. Accordingly, we now took the chance to integrate this idea into the discussion of our findings. (p. 26)

Finally, In your response to the first of the 'Minor issues' raised by R1 you include some new text explaining the poisson GEE models but it looks to me like the old text is still included in the analytic strategy explanation on p. 18? R1 also notes a word missing in line 18 on this page and R2 suggests a careful edit which I agree would be helpful.

> R: Thank you very much for pointing this out. We now changed this section the way we introduced it already in the previous revision. We also added the missing word and took the suggestion of a careful edit reading very serious and asked a third person to proof-read the entire manuscript. Several sentences were identified which were difficult to understand and were now revised for easier comprehension.

These are only minor edits/queries but I think they will improve the manuscript for publication once they are done.

Yours sincerely,
Anne

**Comments of the reviewer:**

Reviewer #1: Unfortunately, I still struggle to follow the analysis in this paper and to understand whether the conclusions are supported by the data, as the inferences seem to derive from the multiple interaction terms, which are difficult to follow. I am unable to comment on the suitability of the statistical models, but here are a few suggestions to improve the reader’s experience:
> Response: We are very grateful for your thorough reading and all the suggest improvements of the paper. Thanks to those comments, we noticed that the manuscript contained several language flaws, was at times difficult to understand and even used misleading language. Thank you!

- Please could you be clearer by what you mean by “The 12-years age range is based on 10 years of observation plus 2 additional years one at the beginning and one at the end of the period which rely on the clustering of the age variable in 2-years bracket” (page 18 line 8-10)
> Response: We understand that this thought was not presented well. We separated now the underlying ideas and revised this sentence to:
“The 12-year age range is based on a 11 years of observation period of the applied data (2004-2014) plus one year due to our categorisation of age groups. As shown in Supplement Table S1, a person born in 1948 is 56 years old at the first observation in 2004. He or she can be traced until age 66 in 2014. Due to the clustering of age in 2-years intervals, a person born one year later would be included in the same health trajectory (age 57 to 67).” (p. 18)

-Please could you correct “To test whether countries differ statistical significance,” (page 18, line 18)?
> R: Apologies for this mistake. We revised this sentence to:
“To test whether countries differ statistically significant, …”

Additionally, we revised the paper regarding more precise language (see later comment) and let the manuscript be proof-read to avoid further mistakes.

- It would help if the authors summarized the key results at very start of the discussion.
> R: We understand that the results are very detailed and appreciate the suggestion to added central findings directly at the beginning of the discussion section. While we also aimed to improve the reading flow of the results, we also took the chance to revise the entire discussion with introducing first some more contextualizing of the study before we presented the three main results in depth. We believe the structure of the discussion is now easier to follow and the main findings are presented more clearly at the beginning of each paragraph. (p. 24)

- Figure 1: currently the panels for England and Germany are of different widths. It would help the comparison if they were the same.
> R: Thank you for pointing this out. We revised Fig. 1 accordingly.

- Figure 2: could a legend be incorporated into the figure, rather than being explained in the note below the figure? Also it would be help if you could see in the figure region which cohorts the lines pertain to.
> R: Again, we appreciate your suggestions to increase easy readability and revised Fig. 2 accordingly.

- Could you explain what you mean by confirmatory bias in page 25 line 14? Why is survivor bias not discussed here?
> R: Please apologize, we have missed to reply in the previous revision to your comment regarding he healthy survivor bias. While we introduced this idea already in section 2c) but missed the opportunity to use it as stronghold/argument for the findings of our study in the discussion. While the idea of “confirmatory bias” (assuming that for CAD papers it is easier to get published if the authors find the expected pattern) would need more space to be introduced, we revised the paragraph and added instead the idea on healthy survivor bias. (p. 26)

- The wording “as differences between educational groups decline both by age and cohort in England, and in Germany educational differences decline only over cohort” in the conclusion is confusing, as educational differences increase in more recent cohorts. It is worth being more precise in language throughout the manuscript so that the message is clear.
> R: Thank you for your thorough reading! We revised this sentence to

“The current study indicates that CAD processes cannot be detected in England and Germany after the age of 50, as differences between educational groups decline by age in England and do not increase significantly in Germany. We found, however, increases in health differences between educational groups for later-born cohorts in both countries, which can be understood as increases in inequality between cohorts, due to educational expansion.“ (p. 30)

Additionally, we noticed that we used a similar wording in the abstract which we revised accordingly.

Finally, we and a third person re-read the entire manuscript to check for similar incoherence in language. This process indicated several sentences over the entire manuscript which were difficult to understand which we were happy to revise.

Reviewer #2: I think that the authors have done a good job responding to the reviewers' comments and have written a very interesting paper. However, although I myself am not a native-English speaker, I encourage the authors to pay attention to errors in the English language (and to odd sentence construction) and to proof-read the article carefully. See, for instance, in the abstract 'more and more'; why is there an acronym if you only mention ADLs once?; should you perhaps add trajectories "of ADLs" among 6 birth cohorts? Or "depending" on p12.
> R: Thank you for putting so much afford in reviewing our paper. We re-read the paper and let a third person proof-read to check for further English mistakes.

Reviewer #3: The authors have addressed my peer review comments, plus other updates to strengthen the manuscript. Great work!
> R: Thank you for helping us to improve the paper!
